# Association between akkermansia and escherichia abundances and glucose metabolism parameters in the gut microbiota of Brazilians

**DOI:** 10.1186/1758-5996-7-S1-A122

**Published:** 2015-11-11

**Authors:** Bianca de Almeida Pititto, Ana Carolina Franco de Moraes, Gabriel da Rocha Fernandes, Everton Padilha Gomes, Alexandre da Costa Pereira, Sandra RG Ferreira

**Affiliations:** 1 UNIFESP, São Paulo, Brazil

## Background

Human body harbors ten times more bacteria than human cells, indicating that microbial communities should play a role for health and diseases. Changes in gut microbiota composition can alter gut barrier, lipopolysaccharides (LPS) translocation and trigger metabolic endotoxemia and insulin resistance. Akkermansia has been associated with a protective effect on the gut barrier by increasing mucus layer. In contrast, Escherichia is a gram-negative bacterium that contains LPS on your surface.

## Objectives

We compared the abundance of Akkermansia and Escherichia in the gut microbiota of individuals stratified according to glucose tolerance and tested associations of their abundances with several biomarkers.

## Materials and methods

This cross-sectional study included 295 individuals divided into normal and abnormal glucose tolerance groups. Abnormal group was defined by the presence of impaired fasting glucose, impaired glucose tolerance or diabetes. The molecular profile of the fecal microbiota was obtained by sequencing V4 region of 16S rRNA gene (Illumina®Miseq). For the purpose of the present analysis, only individuals with biologically significant levels of Akkermansia (>1%) were included in the correlation analyses (N=94).

## Results

From 295 (54.2% women; 49.5±8.4 yrs.; 25.1±4.5 kg/m2), 188 showed normal and 107 abnormal glucose tolerance. As expected, mean values of BMI, plasma glucose, glycated hemoglobin, HOMA-IR and CRP were lower in the normotolerant group (p<0.05). Akkermansia abundance was inversely correlated to CRP and HOMA-IR (r=-0.219 and r=-0.208; p=0.017 and p=0.022, respectively), while the Escherichia abundance showed significant positive correlations to IL-6, CRP and HOMA-IR (r=0.215, r=0.349 and r=0.248; p=0.019 and p<0.001 and p=0.008, respectively).

## Conclusions

The associations of Akkermansia and Escherichia abundances respectively with favorable and unfavorable metabolic profile could suggest a role of these genus in glucose metabolism. This cross-sectional design precludes stablishing cause-effect relationship. Intervention studies are needed to investigate the potential of Akkermansia for the prevention of obesity-related disorders.

